# Are There Causal Associations Between Obsessive-Compulsive Disorder and Cardiometabolic Phenotypes? A Genetic Correlation and Bi-Directional Mendelian Randomization Study

**DOI:** 10.1002/ajmgb.70003

**Published:** 2026-01-13

**Authors:** Robyn E. Wootton, James J. Crowley, Josep Pol-Fuster, Anna Holmberg, Christian Rück, Obsessive-Compulsive Disorder Working Group of the Psychiatric Genomics Consortium, David Mataix-Cols, Lorena Fernández de la Cruz

**Affiliations:** 1 School of Psychological Science, University of Bristol, Bristol, UK; 2 Medical Research Council (MRC) Integrative Epidemiology Unit, Bristol Medical School, University of Bristol, Bristol, UK; 3 Psychiatric Genetic Epidemiology Group, Research Department, Lovisenberg Diaconal Hospital, Oslo, Norway; 4 PsychGen Centre for Genetic Epidemiology and Mental Health, Norwegian Institute of Public Health, Oslo, Norway; 5 Centre for Psychiatry Research, Department of Clinical Neuroscience, Karolinska Institutet & Stockholm Health Care Services, Region Stockholm, Stockholm, Sweden; 6 Department of Genetics, University of North Carolina at Chapel Hill, Chapel Hill, North Carolina, USA; 7 Department of Clinical Sciences, Lund University, Lund, Sweden

**Keywords:** cardiovascular disorders, causality, genetic correlations, Mendelian randomization, metabolic conditions, obsessive-compulsive disorder

## Abstract

In epidemiological studies, obsessive-compulsive disorder (OCD) is robustly associated with increased risk of cardiometabolic disorders, including cardiovascular diseases, type 2 diabetes, and obesity. However, the mechanisms behind these associations are unclear. We conducted genetic correlation analyses to explore shared genetic etiology and bi-directional summary-level Mendelian randomization (MR) to explore potential causal effects between genetic liability to OCD and 14 cardiometabolic phenotypes (e.g., coronary artery disease, blood pressure, body mass index [BMI]). If causal effects were observed, we planned to conduct multivariable MR to explore indirect effects via health behaviors. We found no evidence for genetic correlations between OCD and any of the cardiometabolic phenotypes under study, except for a negative correlation with BMI (rG = −0.123, SE = 0.029, *p* < 0.001). Summary-level MR showed no evidence for causal effects. Therefore, multivariable MR was not conducted. We found limited evidence for shared genetic etiology or causal effects using the largest OCD GWAS to date. However, we were predominantly only powered to detect medium to large effects in the direction of OCD to cardiometabolic traits, leaving the possibility of smaller causal effects existing. Future studies with larger, more representative samples will help to further interpret findings.

## 1 | Introduction

Obsessive-compulsive disorder (OCD) is a chronic mental disorder affecting around 1%–2% of the population (Ruscio et al. 2010). Epidemiological studies have shown a robust link between OCD and morbidity and mortality due to endocrine, metabolic, and circulatory system disorders, such as cardiovascular diseases (CVD), type 2 diabetes, and obesity (Fernández de la Cruz et al. 2024; Isomura et al. 2018, 2021; Chen et al. 2021). However, the mechanisms behind these associations are unclear but likely to be multifactorial, including genetic and environmental factors, such as unhealthy lifestyles and their interaction (Fernández de la Cruz et al. 2022).

Population-based studies using quasi-experimental discordant sibling designs have shown that the associations between OCD and cardiometabolic disorders survive adjustment for unmeasured familial confounding (Fernández de la Cruz et al. 2024; Isomura et al. 2018, 2021). Consistent with these findings, familial coaggregation analyses showed limited evidence for shared familial risk between OCD and CVD, obesity, type 2 diabetes, and hyperlipidemia (Holmberg et al. 2025). Taken together, these epidemiological results seem to suggest that the contribution of shared genetic risk factors to the association between OCD and cardiometabolic disorders is small or negligible.

Along the same lines, the largest OCD genome-wide association study (GWAS) to date, including over 50,000 cases and identifying 30 OCD-associated loci (Strom et al. 2025), did not find significant genetic correlations between OCD and several cardiovascular and metabolic phenotypes, including myocardial infarction, coronary artery disease, type 2 diabetes, cholesterol, and triglycerides. However, the same study found significant negative correlations with hip circumference, body fat, and body mass index (BMI). Thus, the pattern of genetic correlations may differ according to the specific phenotype under study. Moreover, although causal effects are more likely when genetic correlations are identified, the absence of genetic correlation does not preclude the presence of a causal effect (Frei et al. 2019). Here we extend previous genetic correlation analyses to include a broader range of cardiometabolic traits.

A complementary method to further understand the nature of the association between OCD and cardiometabolic outcomes is Mendelian randomization (MR). MR is a genetically informed design for potential causal inference that treats genetic variation as a natural experiment in which individuals are genetically predisposed to higher versus lower mean levels of an exposure during their lifetime (Wootton et al. 2022). Because genetic variants are considered to be allocated randomly before birth, they are relatively independent of environmental factors and established well before onset of disease, minimizing the issues with residual confounding and reverse causation that so often appear in observational studies (Wootton et al. 2022; Byrne et al. 2017). To our knowledge, three MR studies have explored the causal associations between OCD and cardiometabolic outcomes. One study found that OCD was not causally associated with CVD (Yu et al. 2024), while the other two found evidence of a causal negative association between obesity (operationalized as higher BMI or waist-hip ratio) and OCD (Chen et al. 2023; Xiao et al. 2024). These studies focused on one single cardiometabolic phenotype and were limited by the use of older OCD GWAS summary statistics (e.g., International Obsessive Compulsive Disorder Foundation Genetics, OCD Collaborative Genetics Association Studies 2018). With the recent publication of the largest OCD GWAS to date (Strom et al. 2025), and greater variance explained by the genetic instrument, we now have improved power to assess causal effects between OCD and cardiometabolic phenotypes.

This study investigated the potentially causal associations between OCD and 14 cardiometabolic disorders and traits by computing genetic correlations and bi-directional MR. Our study is novel in using the latest summary statistics from the first adequately powered OCD GWAS (Strom et al. 2025) and including a broad range of cardiometabolic phenotypes with publicly available GWAS to explore in greater depth the relationship between OCD and cardiometabolic health. Our specific aims were to: (1) compute genetic correlations to determine the genetic overlap between OCD and cardiometabolic disorders; (2) perform bi-directional univariable MR to test if liability to OCD causally increases cardiometabolic risk, and vice versa; and (3) perform multivariable MR (MVMR) to test if key behaviors (e.g., alcohol consumption, use of medication) mediate effects of liability to OCD on cardiometabolic risk if causal associations were found.

## 2 | Methods

### 2.1 | Data Sources and Measures

#### 2.1.1 | Obsessive-Compulsive Disorder

The most recent GWAS of OCD comprised 53,660 cases and 2,044,417 controls (Strom et al. 2025). Cases were identified either through medical records, clinician assessment, or self-report. Data were obtained from 28 OCD case–control cohorts. A total of 20,427 cases were ascertained by direct clinical assessment or healthcare records and met criteria for OCD as defined by the DSM-5 or ICD-10. The remaining 32,233 cases were obtained from self-report of OCD diagnosis. GWAS was conducted using logistic regression, adjusting for age, sex, and principal components of ancestry. Analyses were restricted to those of European ancestry. There were a total of 30 independent genome-wide significant single nucleoid polymorphisms (SNPs) identified, and the total SNP-based heritability was 6.7%.

#### 2.1.2 | Cardiometabolic Phenotypes

The included 14 cardiometabolic traits were: coronary artery disease, myocardial infarction, heart failure, heart rate variability, triglycerides, total cholesterol, high-density lipoprotein (HDL) cholesterol, low-density lipoprotein (LDL) cholesterol, type 2 diabetes, BMI, ischemic stroke, systolic blood pressure, diastolic blood pressure, and thrombosis.

#### 2.1.3 | Positive Control

We included suicide attempts as a positive control outcome, where we hypothesized a causal effect of genetic liability to OCD on increased risk of suicide attempts (Sidorchuk et al. 2021; Krebs et al. 2021; Tyagi and Bundies 2021). To this end, we used the most recent available GWAS of suicide attempts, comprising 35,786 cases and 779,392 controls of European ancestry (Docherty et al. 2023). As cardiometabolic traits have previously been used as MR instruments and had greater power, we did not employ positive controls in the opposite direction.

### 2.2 | GWAS Summary Statistics

All analyses were conducted using publicly available summary statistics from previously conducted GWAS, summarized in Table 1. We prioritized the largest available GWAS of each trait with the least possible sample overlap as this can bias results toward the confounded estimate when instruments are weak (Burgess et al. 2016). The estimated percentage of overlap, reported in Table 1, was below 1% for all cardiometabolic phenotypes with the exception of thrombosis (2.45%) and heart failure (18.8%). All analyses were conducted in individuals of European ancestry, with the exception of myocardial infarction, where available summary statistics were of mixed ancestry (Nikpay et al. 2015), so we performed a replication using FinnGen data (Kurki et al. 2023) (Note S1). SNP effects for all continuous traits were converted to standardized betas and SNP effects for binary traits were log odds ratios.

### 2.3 | Statistical Analysis

#### 2.3.1 | Genetic Correlations

Genetic correlations were estimated between OCD and each of the cardiometabolic traits using cross-trait LD Score Regression v1.0.1 (Bulik-Sullivan et al. 2015). This method estimates the degree of concordance between effect sizes for the same SNPs across the two traits, while additionally accounting for the degree of linkage disequilibrium (LD) between SNPs. A SNP’s effect size will be inflated, as this effect includes the effects of the SNPs in LD. Therefore, if a trait is polygenic, SNPs with higher LD will show larger effect sizes than those with lower LD. The GWAS estimate for a particular SNP incorporates the effects of all other SNPs that are in LD with that SNP. This information is used to compute an overall genetic correlation between two phenotypes across all SNPs (Bulik-Sullivan et al. 2015).

#### 2.3.2 | MR Analysis Using Summary-Level Data

Summary level (or two-sample) MR was applied to explore evidence for bi-directional causal effects between liability to OCD and a range of cardiometabolic traits. MR utilizes genetic variants as instrumental variables to estimate the causal effect of an exposure (e.g., OCD) on an outcome (e.g., cardiometabolic phenotypes). Genetic variants were identified as those which were associated in previous GWAS at the genome-wide level of significance. Three core assumptions must be satisfied in order to infer causality: (1) the relevance assumption—the genetic instrument must be robustly associated with the exposure; (2) the independence assumption—there must be no confounders of the genetic instrument and the outcome; and (3) the exclusion-restriction assumption—the genetic instrument must only be associated with the outcome via the exposure (Sanderson et al. 2022). The exclusion-restriction assumption can be violated in the presence of horizontal pleiotropy, which occurs when the genetic instruments for the exposure are also associated with confounders or the outcome via other pathways. We use a range of sensitivity analyses (outlined below) to explore the likelihood of assumption violations and bias due to pleiotropy.

All analyses were conducted using the TwoSampleMR package, version 0.6.1 in R version 4.4.0. We used four different MR methods: inverse-variance weighted, MR Egger, weighted median, and weighted mode. Each method makes different assumptions about the presence of pleiotropy and therefore a consistent effect across multiple methods strengthens causal evidence. If a SNP was unavailable in the outcome GWAS summary statistics, then proxy SNPs were searched for with a minimum LD *r*^2^ = 0.8, and palindromic SNPs were aligned if minor allele frequency (MAF) < 0.3. We estimated instrument strength by calculating the mean *F*-statistic across all SNPs, where an *F*-statistic < 10 is considered weak. We calculated the regression dilution *I*^2^_GX_ as an indicator of the suitability of the instrument for MR Egger. We performed Rucker’s *Q*-test of heterogeneity and the MR Egger intercept test to estimate potential directional horizontal pleiotropy. We performed Steiger filtering to check that all genetic variants explained more variance in the exposure than the outcome. If this were not the case, it could suggest potential reverse causation. When there was evidence for causal effects, scatter plots and leave-one-out SNP plots were visually inspected to check for possible outliers.

Where exposures were binary, effect estimates were multiplied by 0.693 so that units can be interpreted as a change per doubling in the odds of the exposure (Burgess and Labrecque 2018). Where outcomes were binary, effect estimates are presented as odds ratios. Where outcomes were continuous, effect estimates are presented as standardized betas.

We estimated the minimum effect size for 80% power in all univariable MR analyses using the mRnd power calculator (Brion et al. 2013). Power calculations did not guide the analyses conducted but were used in effect interpretation.

#### 2.3.3 | Multivariable MR

If evidence for causal effects were observed in MR analyses, we planned to follow up using summary-level MVMR (Sanderson et al. 2019). MVMR is an extension of the MR method where multiple exposures can be included to explore possible indirect pathways. Estimates of one exposure, after conditioning on the other exposure, can reveal whether effects are independent. Where we found evidence for causal effects in univariable MR of liability to OCD on cardiometabolic traits, we planned to investigate whether direct effects were explained through health behaviors: alcohol consumption (Liu et al. 2019), tobacco smoking (Liu et al. 2019; Wootton et al. 2020), physical activity (Wang et al. 2022), BMI (Locke et al. 2015), and diet (Meddens et al. 2021). We planned to conduct analyses using the “MVMR” package in R (Sanderson et al. 2021) using genome-wide summary statistics (a two-sample approach). We planned to estimate *F*-statistics conditional on the other exposure to ensure instruments were of sufficient strength (Sanderson et al. 2021).

## 3 | Results

### 3.1 | Genetic Correlations

**FIGURE 1 |:**
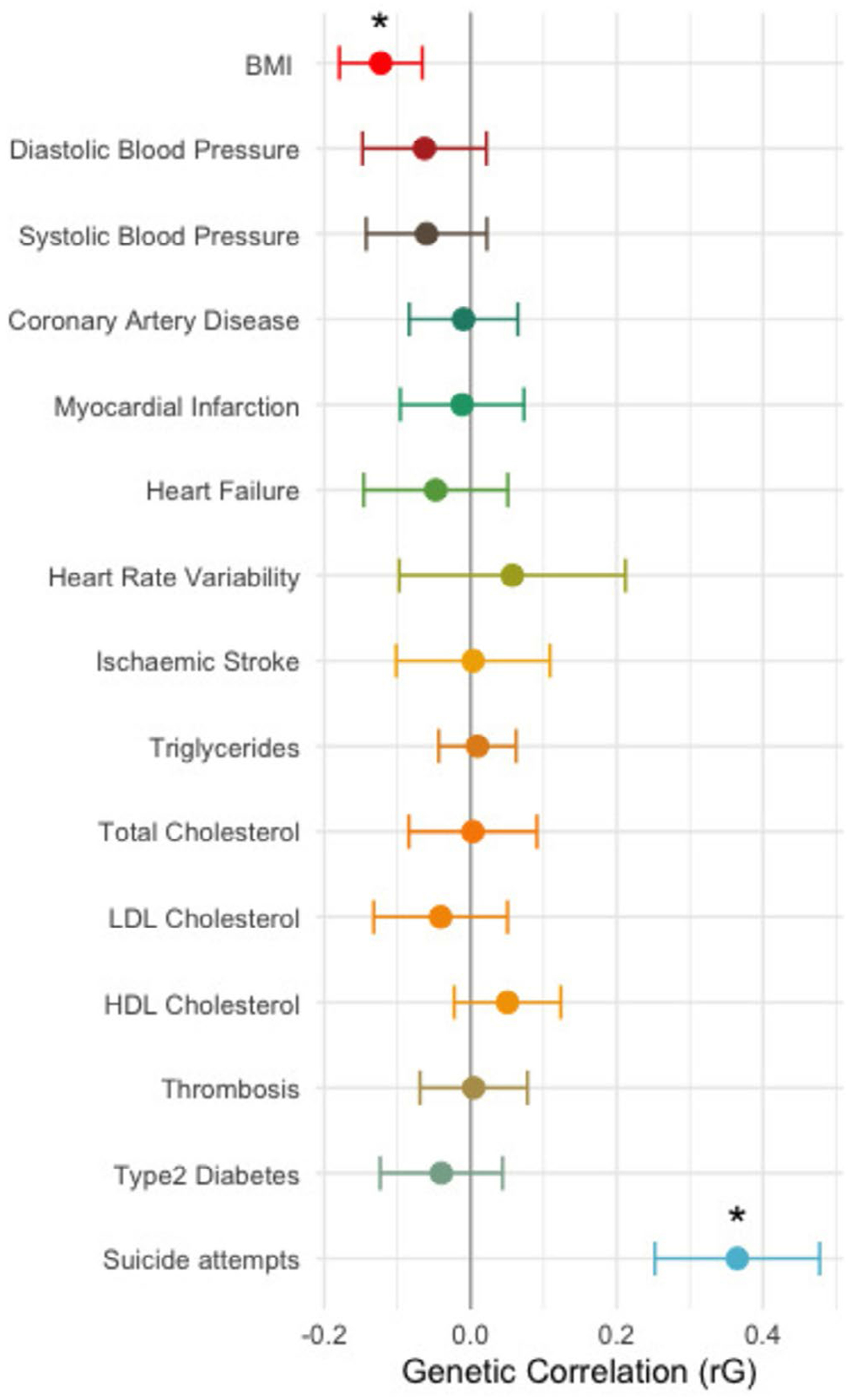
Genetic correlations between OCD and cardiometabolic phenotypes. BMI, body mass index; HDL, high-density lipoprotein; LDL, low-density lipoprotein. * refers to *p* < 0.003 (the Bonferroni corrected *p*-value for 15 tests).

The results of genetic correlations between OCD and cardiometabolic phenotypes are shown in Figure 1. As anticipated, there was evidence for a positive genetic correlation between OCD and our positive control phenotype of suicide attempts (rG = 0.365, SE = 0.058, *p* < 0.001). There was no evidence for a significant overall genetic correlation between OCD and any of the cardiometabolic traits, with the exception of BMI, which was significantly negatively genetically correlated (rG = −0.123, SE = 0.029, *p* < 0.001).

### 3.2 | Mendelian Randomization

#### 3.2.1 | Power Analyses and Instrument Strength

When OCD was the exposure and cardiometabolic phenotypes were the outcome, power calculations demonstrated we only had 80% power to detect medium to large effect sizes in MR analyses, with the exception of power to detect small effects on ischemic stroke (Table S1). In the reverse direction, we had 80% power to detect small effects on OCD when cardiometabolic traits were the exposure, with the exception of ischemic stroke and heart failure, where we only had power to detect large effects (Table S1).

All genetic instruments had sufficient instrument strength to conduct MR analyses with reduced risk of weak instrument bias, as evidenced by a mean *F* statistic > 10 (Table S2).

#### 3.2.2 | Effects of OCD on Cardiometabolic Outcomes

The primary MR method was the inverse-variance weighted method, which assumes balanced horizontal pleiotropy. It was not possible to estimate the MR Egger slope when OCD was the exposure due to evidence of regression dilution (Table S2). Therefore, only weighted median and weighted mode were used as sensitivity methods.

**FIGURE 2 |:**
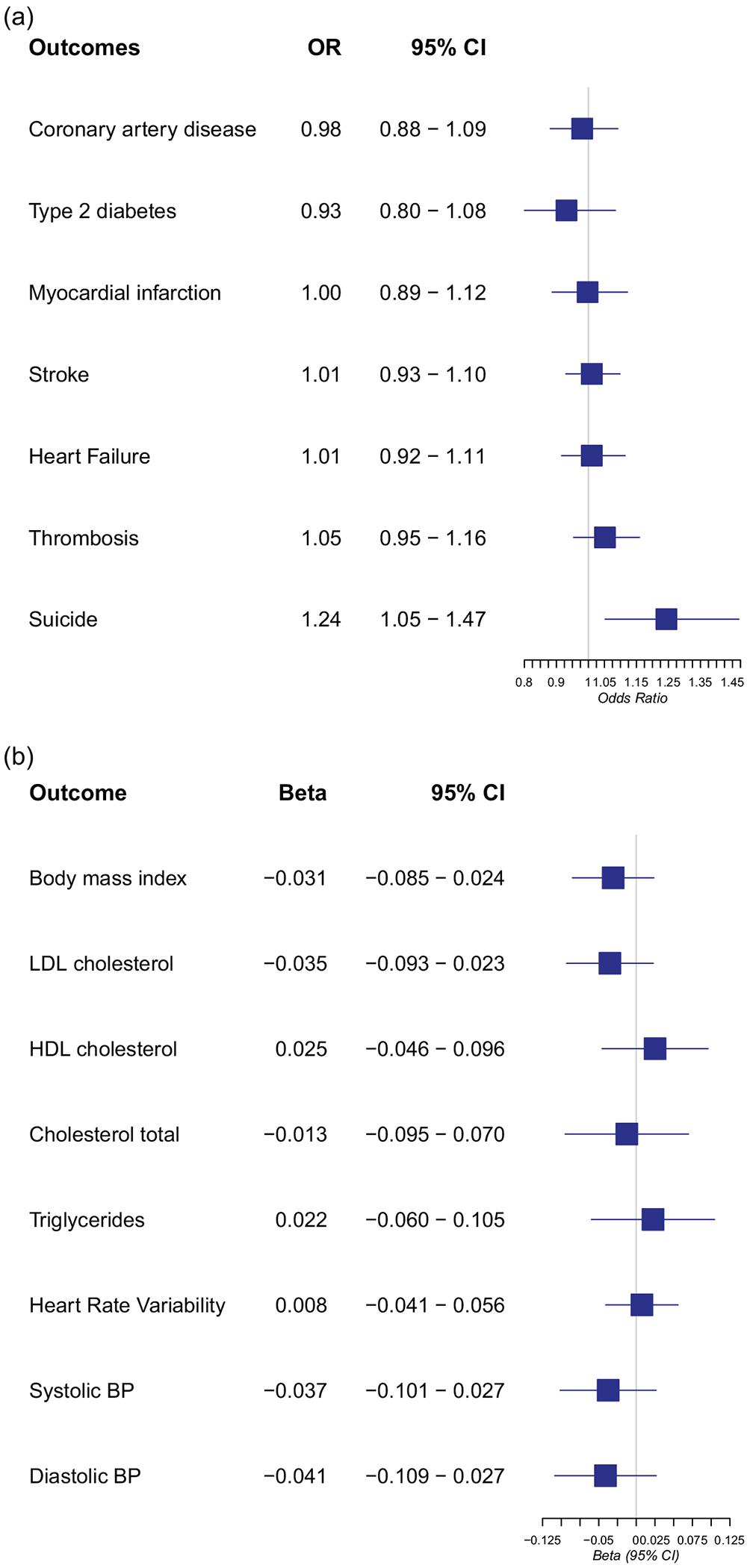
Univariable Mendelian randomization results (inverse variance weighted) for liability to obsessive-compulsive predicting cardiometabolic phenotypes. BP, blood pressure; HDL, high-density lipoprotein; LDL, low-density lipoprotein.

As anticipated, there was evidence to support a causal effect of liability to OCD on our positive control outcome of suicide attempts (Figure 2) such that per doubling in the odds of OCD, there was a 24% increased odds of suicide attempts (95% CI: 5%– 47%, *p* = 0.001). Effect estimates were highly consistent across the sensitivity methods weighted median and weighted mode (Figures S1 and S2). Visual inspection did not suggest results were biased by outlying SNPs (Figures S4 and S5).

MR analyses did not provide evidence to support a causal effect of OCD on any of the cardiometabolic traits (Figure 2), and this was consistent across weighted median and weighted mode sensitivity methods (Figures S1 and S2). There was evidence for significant heterogeneity for several outcomes (thrombosis, BMI, HDL cholesterol, total cholesterol, triglycerides, and blood pressure) (Table S3), which could be the result of pleiotropy. However, the MR Egger intercepts did not suggest that there was significant bias from directional horizontal pleiotropy (with the exception of total cholesterol, triglycerides, and diastolic blood pressure) (Table S4), but these estimates should be interpreted with caution given evidence of regression dilution bias. There was little evidence from Steiger filtering for bias from reverse causation (Table S5), with > 90% of genetic variants explaining more variance in the exposure than the outcome for all traits.

#### 3.2.3 | Effects of Cardiometabolic Traits on OCD Risk

When cardiometabolic traits were the exposures, there was limited evidence of regression dilution bias, with the exception of ischemic stroke (Table S2). Therefore, MR Egger slopes were estimated as a sensitivity test in the majority of analyses, alongside weighted median and weighted mode.

**FIGURE 3 |:**
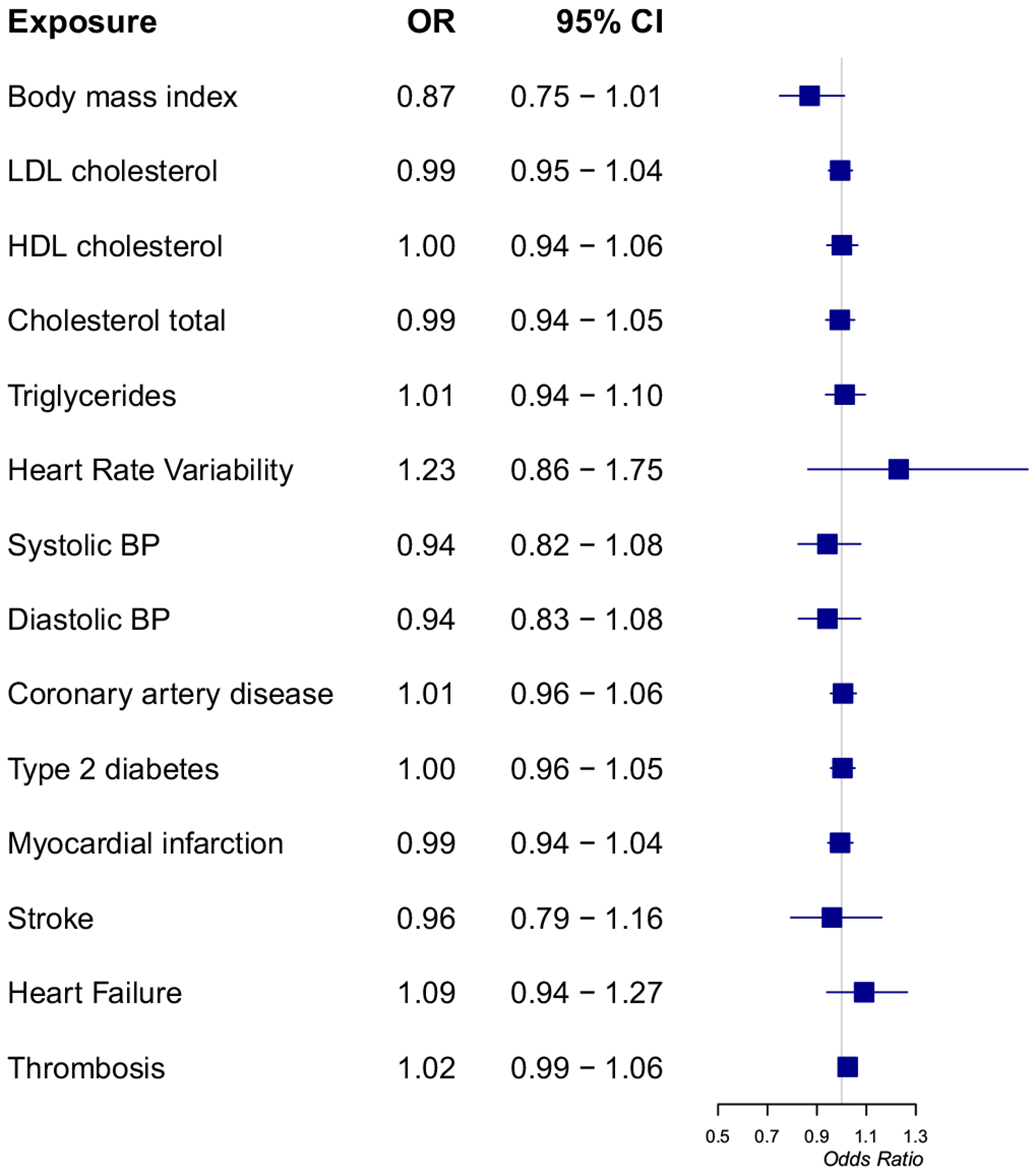
Univariable Mendelian randomization results (inverse variance weighted) for cardiometabolic phenotypes predicting odds of obsessive-compulsive disorder. BP, blood pressure; HDL, high-density lipoprotein; LDL, low-density lipoprotein.

MR analyses did not provide evidence to support a causal effect of any cardiometabolic traits on odds for OCD (Figure 3), and this was consistent across the sensitivity methods (Figure S3). There was evidence for significant heterogeneity across several traits (stroke, BMI, HDL cholesterol, total cholesterol, triglycerides, and blood pressure) (Table S3), but the MR Egger intercept tests suggested no bias from directional horizontal pleiotropy (Table S4). Furthermore, Steiger filtering found no evidence for bias from reverse causation (Table S5), with > 90% of genetic variants explaining more variance in the exposure than the outcome for all traits.

### 3.3 | Multivariable MR

We did not find strong evidence for causal effects of liability to OCD on cardiometabolic outcomes in univariable MR, and therefore MVMR follow-up analyses were not conducted to explore possible mechanisms.

## 4 | Discussion

At the population level, OCD has been shown to be robustly associated with a range of cardiometabolic disorders (Isomura et al. 2018, 2021; Chen et al. 2021). The current study systematically investigated whether these associations could be due to shared genetic etiology by conducting genetic correlations and due to causal effects by conducting bi-directional MR analyses between OCD and 14 cardiovascular and metabolic phenotypes. The results showed that there was no genetic correlation between OCD and any of the cardiometabolic traits, except for BMI, which showed a negative correlation with OCD. Additionally, MR results showed a lack of evidence for causal effects in either direction.

The lack of genetic correlations across a range of cardiometabolic phenotypes replicates the results of the genetic correlations reported in the latest OCD GWAS (Strom et al. 2025), showing no correlation between OCD and 8 cardiovascular phenotypes. Therefore, the observed associations between OCD and cardiometabolic outcomes are unlikely due to shared genetic etiology (at least not from common variants). This is consistent with evidence from previous epidemiological studies using family-based designs, including sibling (Isomura et al. 2018, 2021) and familial coaggregation designs (Holmberg et al. 2025). After controlling for shared genetic and environmental factors, these population-based results suggested that the associations between OCD and cardiometabolic outcomes were largely independent from familial factors, suggesting a possible causal effect. However, our MR analyses showed a lack of evidence to support causal effects in either direction. This could be the result of low statistical power, as we only had power to detect predominantly medium or large effects in the direction of OCD to cardiometabolic phenotypes, and previous evidence suggests some effects might be smaller (Isomura et al. 2018, 2021). Especially given that effects of OCD on cardiometabolic traits are hypothesized to be relatively distal, greater power could be required to detect them.

The negative genetic correlation between OCD and BMI, although consistent with previous studies (Strom et al. 2025; Xiao et al. 2024), is particularly intriguing given that the observed association at the population level is positive (Isomura et al. 2018). Similarly, negative genetic correlations have been described between BMI and schizophrenia (Xiao et al. 2024; Bahrami et al. 2020; Aoki et al. 2022), even when obesity is observed to be up to four times more common in individuals with schizophrenia than in the general population (Correll et al. 2015). One possible explanation could be that diagnosed OCD and sub-threshold OCD traits have different directions of association with BMI. In clinical OCD populations, there is a much higher prevalence of obesity, which might be (to some degree) a result of medication-related weight gain (Albert et al. 2013). However, the GWAS of BMI will only contain OCD cases at population prevalence (1%–2%) (Ruscio et al. 2010), with perhaps even fewer individuals taking OCD medication. Therefore, if the observed positive association between having OCD and higher BMI is due to the consequence of getting a diagnosis and subsequently receiving medication, then this might not be observed in population GWAS of cardiometabolic phenotypes (and consequently in genetic correlations). This might explain why genetic correlations with BMI are not in the expected direction for relatively rare disorders (e.g., schizophrenia and OCD), but are in the expected positive direction for more prevalent disorders (e.g., depression) (Howard et al. 2019). Alternatively, the unexpected association could be due to sample selection, with highly selected samples such as 23andMe and UK Biobank contributing to each of these GWAS respectively. These samples are more highly educated, have a higher socioeconomic status, and tend to be healthier, less likely to be overweight, or have serious mental health conditions (Fry et al. 2017). If both having OCD and being overweight make people less likely to participate in these samples, this results in collider bias, which could induce a spurious negative association between the two traits, highlighting the need for more representative and diverse samples, particularly within genetic research.

Given the lack of evidence for bi-directional causal effects between OCD and cardiometabolic phenotypes, we did not run the MVMR analyses to explore possible mediation through health behaviors such as smoking, unhealthy diet, lack of physical activity, and alcohol consumption. Previous family-based analyses have suggested that these factors may play an important role in the observed associations, as lifestyle behaviors are influenced by non-shared or unique environments. However, these behaviors also have a substantial genetic component (e.g., Maes et al. 2004), which we would expect to pick up in GWAS of OCD and cardiometabolic traits, and subsequently result in a genetic correlation. Furthermore, if these behaviors were on the causal pathway from OCD to cardiometabolic outcomes, we would have expected to observe causal effects in the MR analyses. Given that this is not the case, we think it more likely that results are biased by low statistical power, and future, better powered GWAS of OCD might detect both genetic correlations and causal effects in MR analyses as has been the case for different psychiatric disorders (e.g., depression, anxiety, post-traumatic stress disorder) where greater SNP-heritability has been detected (Yu et al. 2024; Lukas et al. 2025; Li et al. 2022).

Regardless of the mechanisms, individuals with OCD present with an increased risk of morbidity and mortality due to cardiometabolic conditions. Hence, monitoring of their health status and secondary prevention interventions may need to be included in their treatment plans, and the environmental factors that may contribute to the association can be targeted. Lifestyle interventions have been shown to be effective at reducing cardiometabolic-related outcomes in different groups of psychiatric disorders (Bradley et al. 2022), which could also be used in OCD.

## 5 | Strengths and Limitations

This study is the first one to conduct bi-directional MR analyses to systematically evaluate causal associations between OCD and a broad range of cardiometabolic disorders and traits. A handful of previous studies had only focused on a single cardiometabolic phenotype, namely obesity (Chen et al. 2023; Xiao et al. 2024) and CVD (Yu et al. 2024). Additionally, we used the largest OCD GWAS to date (Strom et al. 2025), which identified new OCD genetic risk loci and multiple credible candidate causal genes. For the cardiometabolic phenotypes, we carefully selected publicly available GWAS data to minimize sample overlap with OCD and reduce bias. Finally, to ensure that our OCD genetic instruments were valid, we used a positive control outcome (i.e., suicide attempts) for which, based on previous studies, we hypothesized a positive genetic correlation and a causal association (Sidorchuk et al. 2021; Krebs et al. 2021; Tyagi and Bundies 2021).

The results also need to be interpreted in light of some limitations. First, the OCD GWAS had limited power and, when OCD was the exposure in the MR analyses, we predominantly had power to detect medium to large effect sizes. Therefore, a lack of evidence for causality in the MR analyses should be interpreted with caution, suggesting an absence of evidence rather than evidence of absence. Second, in order to reduce bias from population stratification and to ensure exposure and outcome GWAS summary statistics were from the same underlying population, we restricted to samples from European ancestry populations. As a result, findings may not generalize to other populations. Third, there is known selection bias in several of the largest samples contributing to the GWAS. For example, the UK Biobank sample is highly selected with less than 5% of participants consenting to take part, and those participating being more highly educated and overall healthier than the general UK population (Munafo et al. 2018). If both liability for OCD and liability for cardiometabolic outcomes are negatively associated with participation, then this could induce collider bias, perhaps explaining negative genetic correlations observed. Finally, MR analyses are likely biased by directional horizontal pleiotropy which likely biases estimates away from the null. Given that null effects were observed here, we think that large bias from horizontal pleiotropy is unlikely, especially given that results are relatively consistent across the range of sensitivity methods employed (e.g., MR Egger) which each make different assumptions about the nature of pleiotropy.

## 6 | Conclusions

Our findings align with previous literature using complementary methods and indicate that the association between OCD and cardiometabolic conditions is not likely to be the result of shared genetic liability. Further, we found a lack of evidence for causal effects. However, analyses should be interpreted in light of low statistical power especially in the direction of OCD to cardiometabolic outcomes and should be replicated when larger GWAS of OCD are available.

## Supplementary Material

Supplement

Additional supporting information can be found online in the Supporting Information section. Data S1: Supporting information.

## Figures and Tables

**TABLE 1 |:** Genome-wide summary statistics used in genetic correlations and Mendelian randomization analyses.

Cardiometabolic phenotype	References	Date	*N* case	*N* control	Total *N*	*N* GWS SNPs	Estimated overlap
OCD (with 23andMe)	Strom et al. (2025)	2025	53,660	2,044,417	2,098,077	29	—
OCD (without 23andMe)	Strom et al. (2025)	2025	23,493	1,114,613	1,138,106	—	—
Coronary artery disease	Nikpay et al. (2015)	2015	22,233	64,762	86,995	42	0%
Myocardial infarction	Nikpay et al. (2015)	2015	48,371	123,504	171,875	26	0%
Heart failure	Shah et al. (2020)	2020	47,309	930,014	977,323	12	18.8%
Heart rate variability	Nolte et al. (2017)	2017	—	—	53,174	6	0%
Triglycerides	Willer et al. (2013)	2013	—	—	188,578	54	0.03%
Total cholesterol	Willer et al. (2013)	2013	—	—	188,578	87	0.03%
HDL cholesterol	Willer et al. (2013)	2013	—	—	188,578	87	0.03%
LDL cholesterol	Willer et al. (2013)	2013	—	—	188,578	75	0.03%
Type 2 diabetes	Morris et al. (2012)	2012	12,171	56,862	69,033	33	0.12%
Body mass index	Locke et al. (2015)	2015	—	—	339,224	79	0.06%
Ischemic stroke	Malik et al. (2018)	2018	67,162	454,450	521,612	8	0%
Systolic blood pressure (without UKBB)	Wain et al. (2017)	2017	—	—	150,134	80	0%
Diastolic blood pressure (without UKBB)	Wain et al. (2017)	2017	—	—	150,134	77	0%
Thrombosis	Thibord et al. (2022)	2022	62,879	932,985	995,864	148	2.45%

*Note: N* GWS SNPs = the number of genome-wide significant SNPs for each exposure after restricting to independent variants (kb window = 10,000, *r*^2^ = 0.001). Estimated overlap = Percentage sample overlap estimated at the maximum possible cohort overlap as a percentage of the Strom et al. (2025) OCD GWAS (with 23andMe).

Abbreviations: HDL, high-density lipoprotein; LDL, low-density lipoprotein; OCD, obsessive-compulsive disorder; UKBB, United Kingdom Biobank.

## Data Availability

All GWAS summary statistics are available on the IEU GWAS Repository (https://opengwas.io/), the Psychiatric Genomics Consortium Data Downloads page (https://pgc.unc.edu/for-researchers/download-results/), or via request to the corresponding author of the respective GWAS. Code to reproduce the analyses is available at: https://github.com/robynwootton/OCD_CVD.git.
