# Supplementary material for: Are There Causal Associations Between Obsessive-Compulsive Disorder and Cardiometabolic Phenotypes? A Genetic Correlation and Bi-Directional Mendelian Randomization Study
Source: Am J Med Genet B Neuropsychiatr Genet. Author manuscript; Available in PMC 2026 Jul 13. (PMC13361648; doi:10.1002/ajmgb.70003)

SUPPLEMENTARY MATERIAL

Supplementary Table 1. Power calculations for two-sample Mendelian randomization analysis.

Exposure	Outcome	R^2	Outcome N	Outcome proportion cases	80% Power OR	Effect Size
OCD	Coronary artery disease	0.051%	86,995	0.256	2.15	Large
OCD	Myocardial infarction	0. 051%	171,875	0.281	1.78	Large
OCD	Heart failure	0. 051%	977,323	0.048	1.60	Medium
OCD	Type 2 diabetes	0. 051%	69,033	0.176	2.39	Large
**Exposure**	**Outcome**	**R^2**	**Outcome N**		**80% Power Beta**	**Effect Size**
OCD	Heart rate variability	0. 051%	53,174	-	0.47	Medium
OCD	Triglycerides	0. 051%	188,578	-	0.28	Medium
OCD	Total cholesterol	0. 051%	188,578	-	0.28	Medium
OCD	HDL cholesterol	0. 051%	188,578	-	0.28	Medium
OCD	LDL cholesterol	0. 051%	188,578	-	0.28	Medium
OCD	Body mass index	0. 051%	339,224	-	0.21	Medium
OCD	Ischemic stroke	0. 051%	521,612	-	0.17	Small
OCD	Blood pressure	0. 051%	150,134	-	0.31	Medium
**Exposure**	**Outcome**	**R^2**	**Outcome N**	**Outcome proportion cases**	**80% Power OR**	**Effect Size**
Coronary Artery Disease	OCD	2.88%	985,631	0.038	1.09	Small
Myocardial Infarction	OCD	0.91%	985,631	0.038	1.16	Small

Heart Failure	OCD	0.05%	985,631	0.038	1.67	Large
Type2 Diabetes	OCD	2.69%	985,631	0.038	1.09	Small
Heart Rate Variability	OCD	0.95%	985,631	0.038	1.16	Small
Triglycerides	OCD	4.58%	985,631	0.038	1.07	Small
Total Cholesterol	OCD	6.14%	985,631	0.038	1.06	Small
HDL Cholesterol	OCD	6.79%	985,631	0.038	1.06	Small
LDL Cholesterol	OCD	6.98%	985,631	0.038	1.06	Small
BMI	OCD	1.53%	985,631	0.038	1.12	Small
Ischaemic Stroke	OCD	0.06%	985,631	0.038	1.60	Large
Systolic Blood Pressure	OCD	2.51%	985,631	0.038	1.10	Small
Diastolic Blood Pressure	OCD	2.40%	985,631	0.038	1.10	Small

Note. OCD = Obsessive-compulsive disorder. OR = odds ratios. Where we are broadly defining small as < 1.2 and medium as <1.6 for odds ratios, and small as < 0.2 and medium as < 0.6 for standardized beta coefficients.

Supplementary Table 2. Tests of instrument strength and suitability of the MR Egger method.

Exposure	Outcome	mF	% F stat >10	I^2_{GX} unweighted	I^2_{GX} weighted
OCD	Coronary Artery Disease	35.55	100	0	0
OCD	Type 2 Diabetes	35.80	100	0	0.81
OCD	Myocardial Infarction	35.55	100	0	0
OCD	Stroke	35.55	100	0	0
OCD	Suicide Attempts	35.27	100	0	0
OCD	BMI	35.89	100	0	0
OCD	LDL Cholesterol	35.89	100	0	0
OCD	HDL Cholesterol	35.89	100	0	0
OCD	Total Cholesterol	35.89	100	0	0
OCD	Triglycerides	35.89	100	0	0
OCD	Heart Failure	35.55	100	0	0
OCD	Heart Rate Variability	35.36	100	0	0
OCD	Systolic Blood Pressure	35.27	100	0	0
OCD	Diastolic Blood Pressure	35.27	100	0	0
OCD	Thrombosis	35.55	100	0	0
OCD	Myocardial Infarction (FinnGen)	35.21	100	0	0.10
Coronary Artery Disease	OCD	63.87	100	0.91	0.91
Type 2 Diabetes	OCD	85.87	100	0.92	0.94
Myocardial Infarction	OCD	61.78	100	0.90	0.90
Stroke	OCD	41.83	100	0	0
BMI	OCD	57.72	100	0.86	0.83
LDL Cholesterol	OCD	181.54	100	0.99	0.99
HDL Cholesterol	OCD	149.95	100	0.98	0.98
Total Cholesterol	OCD	140.45	100	0.98	0.98
Triglycerides	OCD	156.59	100	0.98	0.98
Heart Failure	OCD	41.08	100	0.83	0.20
Heart Rate Variability	OCD	57.21	100	0.80	0.67
Systolic Blood Pressure	OCD	47.59	100	0.72	0.53
Diastolic Blood Pressure	OCD	45.63	100	0.75	0.59
Thrombosis	OCD	122.86	100	0.98	0.98
Myocardial Infarction (FinnGen)	OCD	67.13	100	0.87	0.92

Supplementary Table 3. Cochran's Q estimate of heterogeneity in SNP effects.

Exposure	Outcome	Method	Q	DF	P-value
OCD	Coronary artery disease	MR Egger	45.06	28	0.02
		Inverse variance weighted	45.51	29	0.03
OCD	Type 2 diabetes	MR Egger	29.67	24	0.20
		Inverse variance weighted	30.24	25	0.22
OCD	Myocardial infarction	MR Egger	44.89	28	0.02
		Inverse variance weighted	44.90	29	0.03
OCD	Stroke	MR Egger	26.47	28	0.55
		Inverse variance weighted	26.77	29	0.58
OCD	Heart Failure	MR Egger	54.18	28	0.002
		Inverse variance weighted	55.66	29	0.002
OCD	Thrombosis	MR Egger	89.19	28	<0.001
		Inverse variance weighted	96.28	29	<0.001
OCD	Suicide	MR Egger	87.60	26	<0.001
		Inverse variance weighted	90.04	27	<0.001
OCD	Myocardial infarction (FinnGen)	MR Egger	37.31	27	0.09
		Inverse variance weighted	37.41	28	0.11
OCD	Body mass index	MR Egger	64.81	24	<0.001
		Inverse variance weighted	64.84	25	<0.001
OCD	LDL cholesterol	MR Egger	32.34	24	0.12
		Inverse variance weighted	38.07	25	0.05
OCD	HDL cholesterol	MR Egger	57.15	24	<0.001
		Inverse variance weighted	67.29	25	<0.001
OCD	Cholesterol total	MR Egger	54.87	24	<0.001
		Inverse variance weighted	81.16	25	<0.001
OCD	Triglycerides	MR Egger	64.45	24	<0.001
		Inverse variance weighted	94.85	25	<0.001
OCD	Heart Rate Variability	MR Egger	13.38	13	0.42
		Inverse variance weighted	13.47	14	0.49
OCD	Systolic BP	MR Egger	91.26	26	<0.001
		Inverse variance weighted	112.99	27	<0.001
OCD	Diastolic BP	MR Egger	77.61	26	<0.001
		Inverse variance weighted	107.66	27	<0.001
Coronary artery disease	OCD	MR Egger	43.12	34	0.14
		Inverse variance weighted	44.46	35	0.13
Type 2 diabetes	OCD	MR Egger	37.568	26	0.07
		Inverse variance weighted	40.09	27	0.05
Myocardial infarction	OCD	MR Egger	19.98	22	0.58
		Inverse variance weighted	20.66	23	0.60
Stroke	OCD	MR Egger	10.02	6	0.12

		Inverse variance weighted	14.68	7	0.04
Heart Failure	OCD	MR Egger	11.56	8	0.17
		Inverse variance weighted	11.58	9	0.24
Thrombosis	OCD	MR Egger	129.55	114	0.15
		Inverse variance weighted	129.61	115	0.17
Myocardial infarction (FinnGen)	OCD	MR Egger	10.27	9	0.33
		Inverse variance weighted	10.66	10	0.38
Body mass index	OCD	MR Egger	135.60	71	<0.001
		Inverse variance weighted	136.91	72	<0.001
LDL cholesterol	OCD	MR Egger	85.46	68	0.07
		Inverse variance weighted	87.07	69	0.07
HDL cholesterol	OCD	MR Egger	115.26	84	0.01
		Inverse variance weighted	118.19	85	0.01
Cholesterol total	OCD	MR Egger	107.79	79	0.01
		Inverse variance weighted	108.09	80	0.02
Triglycerides	OCD	MR Egger	72.97	54	0.04
		Inverse variance weighted	77.17	55	0.03
Heart Rate Variability	OCD	MR Egger	0.49	3	0.92
		Inverse variance weighted	0.71	4	0.95
Systolic BP	OCD	MR Egger	97.87	68	0.01
		Inverse variance weighted	98.79	69	0.01
Diastolic BP	OCD	MR Egger	103.51	67	0.003
		Inverse variance weighted	104.58	68	0.003

Supplementary Table 4. MR Egger intercept estimates showing evidence for bias from directional horizontal pleiotropy.

Exposure	Outcome	MR Egger Intercept	P-value
OCD	Coronary artery disease	0.01 (-0.027, 0.046)	0.60
OCD	Type 2 diabetes	-0.018 (-0.07, 0.034)	0.50
OCD	Myocardial infarction	-0.002 (-0.041, 0.038)	0.94
OCD	Stroke	-0.008 (-0.037, 0.021)	0.59
OCD	Heart Failure	-0.014 (-0.046, 0.018)	0.39
OCD	Thrombosis	-0.023 (-0.053, 0.007)	0.15
OCD	Suicide	-0.027 (-0.09, 0.035)	0.40
OCD	Myocardial infarction (FinnGen)	-0.008 (-0.067, 0.051)	0.79
OCD	Body mass index	-0.001 (-0.019, 0.017)	0.92
OCD	LDL cholesterol	-0.017 (-0.034, -0.001)	0.05
OCD	HDL cholesterol	-0.021 (-0.041, -0.001)	0.05
OCD	Cholesterol total	-0.036 (-0.057, -0.015)	0.002
OCD	Triglycerides	-0.036 (-0.057, -0.015)	0.003
OCD	Heart Rate Variability	0.004 (-0.019, 0.026)	0.77
OCD	Systolic BP	0.028 (0.006, 0.05)	0.02
OCD	Diastolic BP	0.036 (0.014, 0.058)	0.004
Coronary artery disease	OCD	0.006 (-0.006, 0.018)	0.31
Type 2 diabetes	OCD	0.01 (-0.005, 0.026)	0.20
Myocardial infarction	OCD	0.005 (-0.007, 0.018)	0.42
Stroke	OCD	-0.067 (-0.146, 0.012)	0.15
Heart Failure	OCD	0.002 (-0.029, 0.032)	0.91
Thrombosis	OCD	-0.001 (-0.005, 0.004)	0.83
Myocardial infarction (FinnGen)	OCD	-0.008 (-0.037, 0.02)	0.58
Body mass index	OCD	-0.005 (-0.016, 0.007)	0.41
LDL cholesterol	OCD	-0.003 (-0.008, 0.002)	0.26
HDL cholesterol	OCD	0.004 (-0.001, 0.009)	0.15
Cholesterol total	OCD	0.001 (-0.004, 0.007)	0.64
Triglycerides	OCD	-0.006 (-0.012, 0.001)	0.08
Heart Rate Variability	OCD	-0.012 (-0.061, 0.038)	0.67
Systolic BP	OCD	-0.006 (-0.019, 0.008)	0.43
Diastolic BP	OCD	-0.006 (-0.019, 0.008)	0.41

Supplementary Table 5. Results of Steiger filtering tests to detect possible evidence of reverse causation.

Exposure	Outcome	N False	N True	% True
OCD	Coronary Artery Disease	0	30	100
OCD	Type 2 Diabetes	1	25	96.15
OCD	Myocardial Infarction	1	29	96.67
OCD	Stroke	0	30	100
OCD	Heart Failure	0	30	100
OCD	BMI	0	26	100
OCD	LDL Cholesterol	0	26	100
OCD	HDL Cholesterol	0	26	100
OCD	Total Cholesterol	1	25	96.15
OCD	Heart Rate Variability	0	15	100
OCD	Triglycerides	1	25	96.15
OCD	Systolic Blood Pressure	2	26	92.86
OCD	Diastolic Blood Pressure	2	26	92.86
OCD	Thrombosis	0	30	100
Coronary Artery Disease	OCD	0	36	100
Type 2 Diabetes	OCD	0	28	100
Myocardial Infarction	OCD	0	24	100
Stroke	OCD	0	8	100
Heart Failure	OCD	0	10	100
BMI	OCD	0	73	100
LDL Cholesterol	OCD	0	70	100
HDL Cholesterol	OCD	0	86	100
Total Cholesterol	OCD	0	81	100
Heart Rate Variability	OCD	0	5	100
Triglycerides	OCD	0	56	100
Systolic Blood Pressure	OCD	0	70	100
Diastolic Blood Pressure	OCD	0	69	100
Thrombosis	OCD	1	115	99.14

Supplementary Figure 1. Full univariable Mendelian randomization results for liability to obsessive-compulsive disorder predicting binary cardiometabolic traits.

Outcome	Method	Odds Ratio	95% CI	P−Value
CAD	Inverse variance weighted	0.98	(0.88 , 1.09)	0.609
	Weighted median	0.96	(0.85 , 1.09)	0.381
	Weighted mode	0.93	(0.74 , 1.19)	0.427
Type 2 Diabetes	Inverse variance weighted	0.93	(0.80 , 1.08)	0.187
	Weighted median	0.85	(0.69 , 1.04)	0.026
	Weighted mode	0.84	(0.61 , 1.17)	0.152
Myocardial infarction	Inverse variance weighted	1.00	(0.89 , 1.12)	0.952
	Weighted median	0.99	(0.86 , 1.13)	0.762
	Weighted mode	0.95	(0.74 , 1.22)	0.565
Stroke	Inverse variance weighted	1.01	(0.93 , 1.10)	0.718
	Weighted median	0.99	(0.89 , 1.11)	0.823
	Weighted mode	0.97	(0.78 , 1.20)	0.679
Heart Failure	Inverse variance weighted	1.01	(0.92 , 1.11)	0.760
	Weighted median	0.97	(0.87 , 1.07)	0.335
	Weighted mode	0.96	(0.78 , 1.18)	0.561
Thrombosis	Inverse variance weighted	1.05	(0.95 , 1.16)	0.145
	Weighted median	1.02	(0.93 , 1.12)	0.492
	Weighted mode	0.98	(0.83 , 1.15)	0.716
Suicide Attempts	Inverse variance weighted	1.24	(1.05 , 1.47)	0.000
	Weighted median	1.24	(1.07 , 1.44)	0.000
	Weighted mode	1.21	(0.88 , 1.67)	0.095

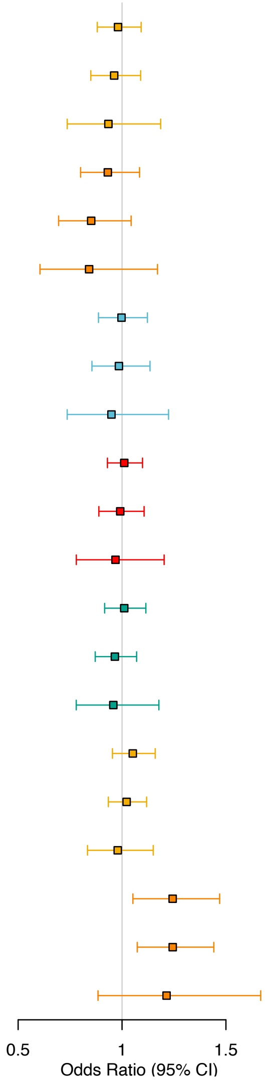

Supplementary Figure 2. Full univariable Mendelian randomization results for liability to obsessive-compulsive disorder predicting continuous cardiometabolic traits.

Outcome	Method	Beta	95% CI	P–Value
BMI	Inverse variance weighted	−0.031	(−0.085 , 0.024)	0.109
	Weighted median	−0.019	(−0.071 , 0.033)	0.295
	Weighted mode	−0.010	(−0.108 , 0.089)	0.788
LDL Cholesterol	Inverse variance weighted	−0.035	(−0.093 , 0.023)	0.086
	Weighted median	−0.025	(−0.094 , 0.044)	0.307
	Weighted mode	−0.021	(−0.136 , 0.095)	0.620
HDL Cholesterol	Inverse variance weighted	0.025	(−0.046 , 0.096)	0.314
	Weighted median	−0.007	(−0.077 , 0.063)	0.777
	Weighted mode	−0.019	(−0.185 , 0.146)	0.744
Total Cholesterol	Inverse variance weighted	−0.013	(−0.095 , 0.070)	0.666
	Weighted median	−0.035	(−0.104 , 0.034)	0.154
	Weighted mode	−0.038	(−0.155 , 0.080)	0.375
Triglycerides	Inverse variance weighted	0.022	(−0.060 , 0.105)	0.441
	Weighted median	−0.008	(−0.076 , 0.060)	0.729
	Weighted mode	−0.013	(−0.139 , 0.112)	0.771
Heart Rate Variability	Inverse variance weighted	0.008	(−0.041 , 0.056)	0.655
	Weighted median	−0.015	(−0.086 , 0.056)	0.543
	Weighted mode	−0.043	(−0.171 , 0.085)	0.358
Systolic BP	Inverse variance weighted	−0.037	(−0.101 , 0.027)	0.098
	Weighted median	−0.050	(−0.098 , −0.001)	0.004
	Weighted mode	−0.051	(−0.134 , 0.031)	0.089
Diastolic BP	Inverse variance weighted	−0.041	(−0.109 , 0.027)	0.087
	Weighted median	−0.045	(−0.096 , 0.005)	0.011
	Weighted mode	−0.055	(−0.159 , 0.049)	0.147

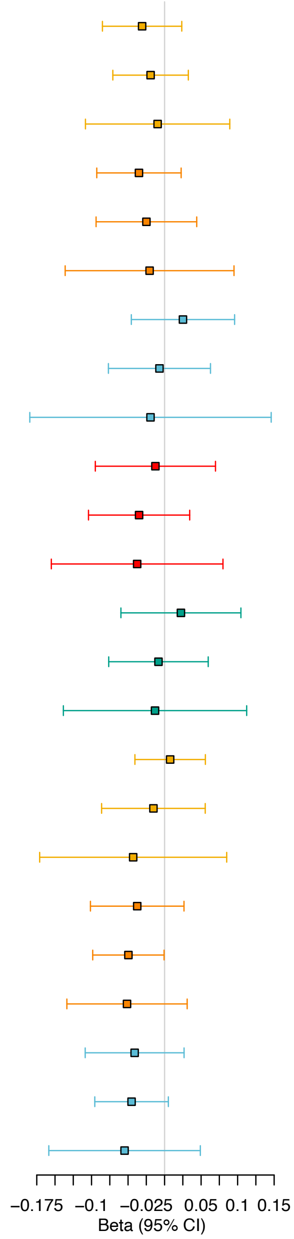

Supplementary Figure 3a. Full univariable Mendelian randomization results for continuous cardiometabolic traits predicting liability to obsessive-compulsive disorder.

Exposure	Method	Odds Ratio	95% CI	P–Value
Body mass index	Inverse variance weighted	0.87	(0.75 , 1.01)	0.068
Body mass index	MR Egger	1.03	(0.67 , 1.57)	0.894
Body mass index	Weighted median	0.89	(0.75 , 1.06)	0.194
Body mass index	Weighted mode	0.89	(0.67 , 1.18)	0.403
LDL cholesterol	Inverse variance weighted	0.99	(0.95 , 1.04)	0.806
LDL cholesterol	MR Egger	1.02	(0.96 , 1.09)	0.574
LDL cholesterol	Weighted median	1.03	(0.97 , 1.10)	0.337
LDL cholesterol	Weighted mode	1.01	(0.95 , 1.07)	0.797
HDL cholesterol	Inverse variance weighted	1.00	(0.94 , 1.06)	0.977
HDL cholesterol	MR Egger	0.94	(0.86 , 1.04)	0.263
HDL cholesterol	Weighted median	0.97	(0.89 , 1.06)	0.538
HDL cholesterol	Weighted mode	0.98	(0.91 , 1.05)	0.549
Cholesterol total	Inverse variance weighted	0.99	(0.94 , 1.05)	0.804
Cholesterol total	MR Egger	0.98	(0.89 , 1.07)	0.606
Cholesterol total	Weighted median	1.02	(0.94 , 1.10)	0.719
Cholesterol total	Weighted mode	1.00	(0.92 , 1.08)	0.989
Triglycerides	Inverse variance weighted	1.01	(0.94 , 1.10)	0.760
Triglycerides	MR Egger	1.11	(0.98 , 1.26)	0.118
Triglycerides	Weighted median	1.05	(0.94 , 1.16)	0.390
Triglycerides	Weighted mode	1.03	(0.93 , 1.13)	0.605
Heart Rate Variability	Inverse variance weighted	1.23	(0.86 , 1.75)	0.250
Heart Rate Variability	MR Egger	1.67	(0.44 , 6.27)	0.503
Heart Rate Variability	Weighted median	1.31	(0.87 , 1.98)	0.192
Heart Rate Variability	Weighted mode	1.33	(0.79 , 2.24)	0.343
Systolic BP	Inverse variance weighted	0.94	(0.82 , 1.08)	0.383
Systolic BP	MR Egger	1.14	(0.70 , 1.87)	0.596
Systolic BP	Weighted median	0.95	(0.79 , 1.13)	0.557
Systolic BP	Weighted mode	0.85	(0.56 , 1.28)	0.442
Diastolic BP	Inverse variance weighted	0.94	(0.83 , 1.08)	0.382
Diastolic BP	MR Egger	1.13	(0.72 , 1.78)	0.592
Diastolic BP	Weighted median	0.97	(0.83 , 1.14)	0.702
Diastolic BP	Weighted mode	0.91	(0.63 , 1.31)	0.622

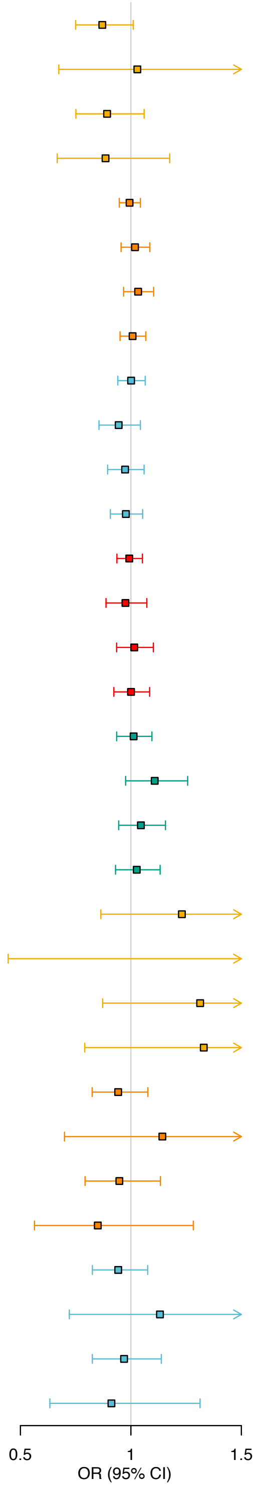

Supplementary Figure 3b. Full univariable Mendelian randomization results for binary cardiometabolic traits predicting liability to obsessive-compulsive disorder.

Exposure	Method	Odds Ratio	95% CI	P–Value
Coronary artery disease	Inverse variance weighted	1.01	(0.96 , 1.06)	0.751
Coronary artery disease	MR Egger	0.97	(0.86 , 1.09)	0.433
Coronary artery disease	Weighted median	0.97	(0.91 , 1.04)	0.239
Coronary artery disease	Weighted mode	0.97	(0.90 , 1.04)	0.261
Type 2 diabetes	Inverse variance weighted	1.00	(0.96 , 1.05)	0.843
Type 2 diabetes	MR Egger	0.95	(0.83 , 1.08)	0.259
Type 2 diabetes	Weighted median	0.98	(0.92 , 1.05)	0.446
Type 2 diabetes	Weighted mode	0.95	(0.88 , 1.03)	0.088
Myocardial infarction	Inverse variance weighted	0.99	(0.94 , 1.04)	0.720
Myocardial infarction	MR Egger	0.96	(0.85 , 1.09)	0.378
Myocardial infarction	Weighted median	0.97	(0.91 , 1.05)	0.304
Myocardial infarction	Weighted mode	0.97	(0.90 , 1.05)	0.245
Stroke	Inverse variance weighted	0.96	(0.79 , 1.16)	0.552
Stroke	Weighted median	0.99	(0.82 , 1.19)	0.833
Stroke	Weighted mode	1.04	(0.80 , 1.36)	0.682
Heart Failure	Inverse variance weighted	1.09	(0.94 , 1.27)	0.096
Heart Failure	MR Egger	1.07	(0.67 , 1.71)	0.688
Heart Failure	Weighted median	1.05	(0.87 , 1.26)	0.473
Heart Failure	Weighted mode	0.98	(0.68 , 1.40)	0.853
Thrombosis	Inverse variance weighted	1.02	(0.99 , 1.06)	0.046
Thrombosis	MR Egger	1.03	(0.97 , 1.09)	0.159
Thrombosis	Weighted median	1.02	(0.96 , 1.07)	0.437
Thrombosis	Weighted mode	1.02	(0.96 , 1.07)	0.391

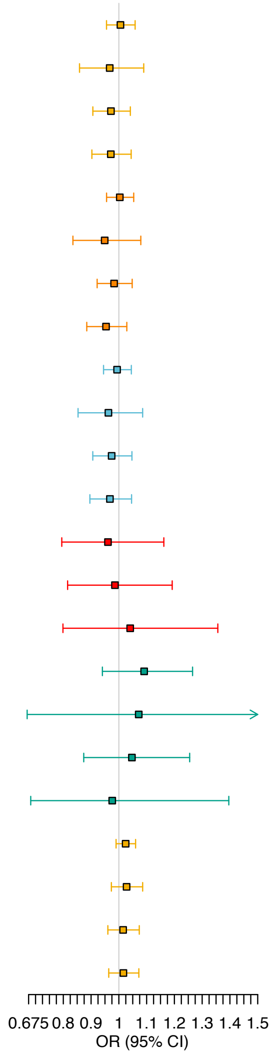

Supplementary Figure 4. Scatter plot of SNP-exposure effects on obsessive-compulsive disorder against SNP-outcome effects on suicide attempts.

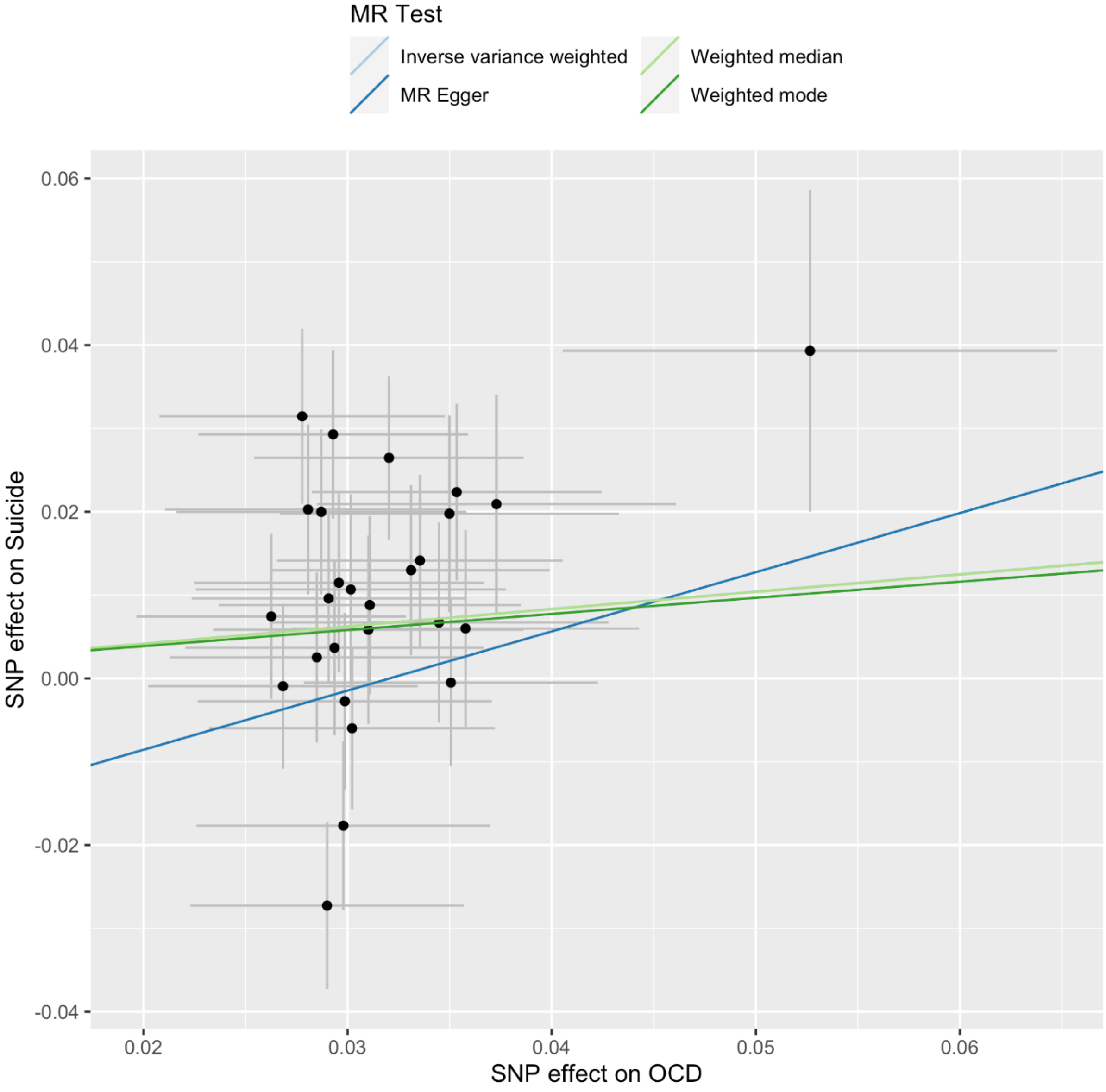

Supplementary Figure 5. Leave-one-out SNP plot of obsessive-compulsive disorder on suicide attempts.

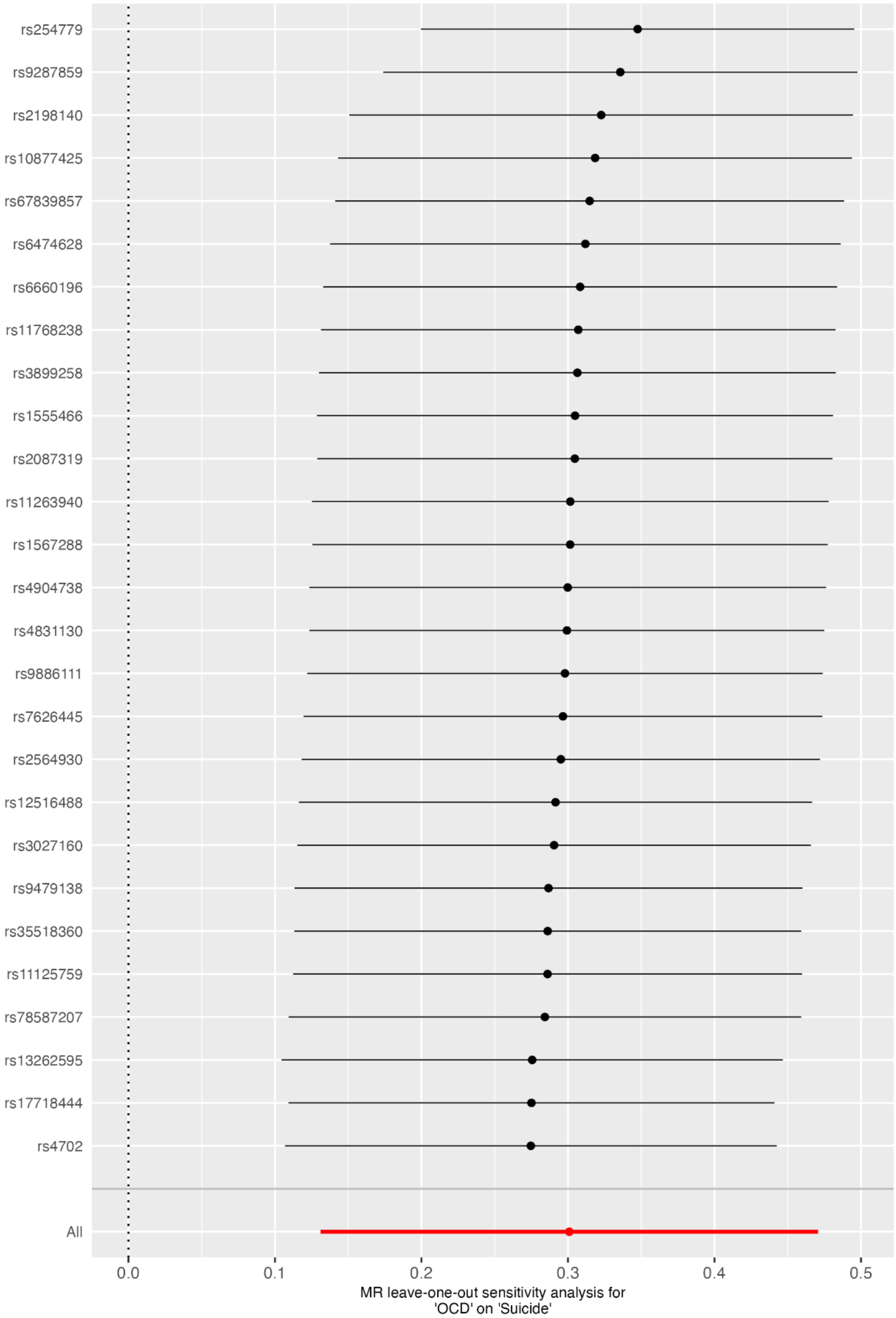

Supplementary Note S1. Sensitivity test due to mixed ancestry summary statistics for myocardial infarction.

Nikpay et al.'s (21) GWAS of myocardial infarction (MI) contains >70% European ancestry participants, and the European ancestry only effect sizes were not available. Therefore, we conducted a sensitivity analysis using the myocardial infarction GWAS summary statistics from the FinnGen consortium (22) (details available at: https://gwas.mrcieu.ac.uk/datasets/finn-b-I9_MI/).

In the figures below, we plot the results of the Nikpay et al.'s (21) GWAS against the 2021 FinnGen consortium results (22), which show incredibly high concordance. First (Figure S6A), when MI is the exposure, odds of experiencing OCD were highly consistent. Second (Figure S6B), when OCD was the exposure and MI was the outcome, again with highly consistent results.

In conclusion, given the high concordance between results using both sets of summary statistics, we felt confident that results were not substantially biased by differences in ancestry of underlying GWAS populations.

Figure S6A.

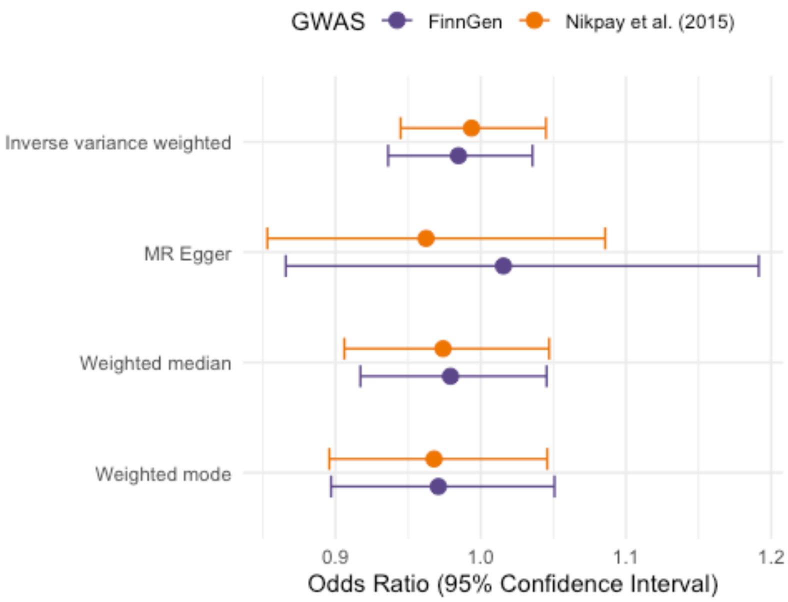

Figure S6B.

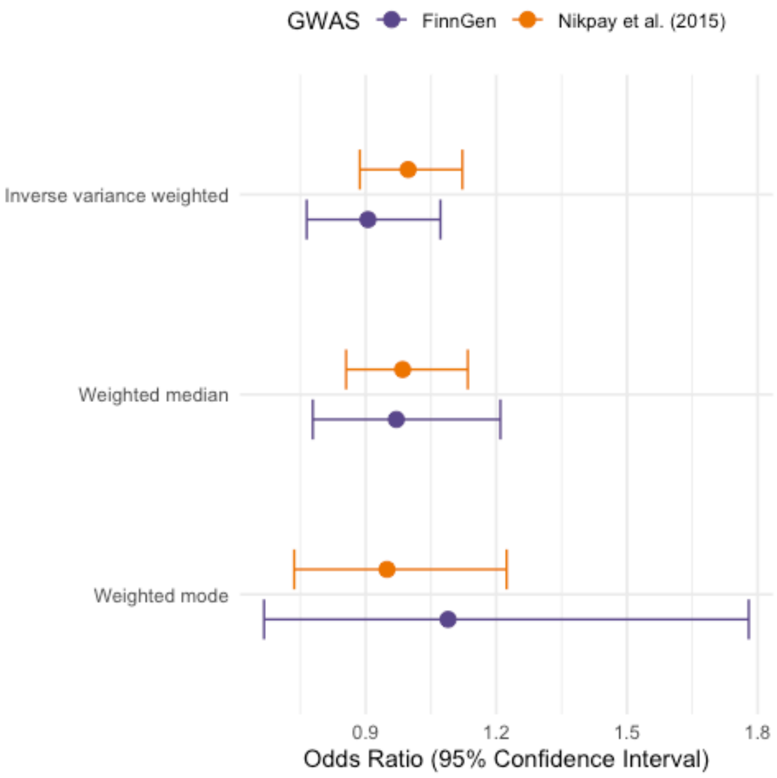

Supplement: Supplement [file NIHMS2186071-supplement-Supplement.pdf]
